# Enhanced Large-Scale Production of *Hahella chejuensis*-Derived Prodigiosin and Evaluation of Its Bioactivity

**DOI:** 10.4014/jmb.2109.09039

**Published:** 2021-10-14

**Authors:** Yu-jin Jeong, Hyun Ju Kim, Suran Kim, Seo-Young Park, HyeRan Kim, Sekyoo Jeong, Sang Jun Lee, Moo-Seung Lee

**Affiliations:** 1 Environmental Diseases Research Center, Korea Research Institute of Bioscience and Biotechnology, Daejeon 34141, Republic of Korea; 2 Department of Systems Biotechnology, Chung-Ang University, Anseong 17546, Republic of Korea; 3 Plant Systems Engineering Research Center, Korea Research Institute of Bioscience and Biotechnology, Daejeon 34141, Republic of Korea; 4 Research Division, Incospharm Corp., Daejeon 34036, Republic of Korea; 5 Department of Biomolecular Science, KRIBB School of Bioscience, Korea University of Science and Technology (UST), Daejeon 34113, Republic of Korea

**Keywords:** *Hahella chejuensis*, prodigiosin, large-scale production, fermentation

## Abstract

Prodigiosin as a high-valued compound, which is a microbial secondary metabolite, has the potential for antioxidant and anticancer effects. However, the large-scale production of functionally active *Hahella chejuensis*-derived prodigiosin by fermentation in a cost-effective manner has yet to be achieved. In the present study, we established carbon source-optimized medium conditions, as well as a procedure for producing prodigiosin by fermentation by culturing *H. chejuensis* using 10 L and 200 L bioreactors. Our results showed that prodigiosin productivity using 250 ml flasks was higher in the presence of glucose than other carbon sources, including mannose, sucrose, galactose, and fructose, and could be scaled up to 10 L and 200 L batches. Productivity in the glucose (2.5 g/l) culture while maintaining the medium at pH 6.89 during 10 days of cultivation in the 200 L bioreactor was measured and increased more than productivity in the basal culture medium in the absence of glucose. Prodigiosin production from 10 L and 200 L fermentation cultures of *H. chejuensis* was confirmed by high-performance liquid chromatography (HPLC) and liquid chromatography&ndash;mass spectrometry (LC&ndash;MS) analyses for more accurate identification. Finally, the anticancer activity of crude extracted prodigiosin against human cancerous leukemia THP-1 cells was evaluated and confirmed at various concentrations. Conclusively, we demonstrate that culture conditions for *H. chejuensis* using a bioreactor with various parameters and ethanol-based extraction procedures were optimized to mass-produce the marine bacterium-derived high purity prodigiosin associated with anti-cancer activity.

## Introduction

Prodigiosin from the prodiginines family is a typical secondary metabolite produced during the microbial idiophase, and is a red-pigmented tripyrrole compound produced by many different bacteria, such as *Streptomyces coelicolor*, *Janthinobacterium lividum*, *Alteromonas rubra*, *Hahella chejuensis*, *Serratia marcescens*, *Rugamonas rubra*, *Streptomyces fusant* NRCF69, *Vibrio psychroerythrus*, *Serratia rubidaea*, and *Streptoverticillium rubrireticuli* [1, 2]. Among these bacteria, *S. marcescens* is the most commonly utilized microbe for prodigiosin production. In *S. marcescens*, prodigiosin bearing the chemical formula C_20_H_25_N_3_O has a tripyrrole molecular configuration with pyrrole (A ring), 3-methoxypyrrole (B ring), and 2-methyl-3-pentylpyrrole (C ring); and two essential intermediate metabolites, 2-methyl-3-*n*-amylpyrrole and 4-methoxy-2,2′-bipyrrole-5-carbaldehyde, are synthesized via a bifurcated pathway. The metabolites 2-methyl-3-*n*-amylpyrrole and 4-methoxy-2,2′-bipyrrole-5-carbaldehyde are eventually assembled by key enzymes to form prodigiosin [3, 4]. This bacterial pigment has numerous biological activities, including antioxidant, algicidal, antibacterial, anti-inflammatory, immunosuppressant, and anticancer activities [5, 6]. Aside from the many reported uses of *S. marcescens*-derived prodigiosin as a food colorant or in candles and cosmetics, we previously demonstrated that prodigiosin produced by *H. chejuensis* can reduce UV-induced reactive oxygen species production, the pro-inflammatory response, and cytotoxicity in irradiated human keratinocytes [7], suggesting potential for cosmetic ingredient development.

These beneficial bioactivities of prodigiosin and its many potential applications have received substantial attention with a dramatic increase in the industrial development of prodigiosin biosynthesis, extraction, and large-scale bioactive compound production via bacterial fermentation [5, 8, 9]. Although tremendous efforts have been made to produce prodigiosin, *S. marcescens* is the species most widely utilized to maximize productivity at a low cost [10, 11]. For increased production of prodigiosin from *S. marcescens* via microbial fermentation, various types of selective media for culturing bacteria have been investigated during the last few decades to vary growth conditions, pH, temperature, carbon and nitrogen sources, and sodium chloride concentrations [12, 13], also reviewed in [14], indicating that changing a single factor or parameter during cell growth was not sufficient to significantly improve productivity. However, no developments have been established and reported for the large-scale fermentative production of *H. chejuensis*-derived prodigiosin, including culture condition optimization or scaling up from a 10 L to a 200 L bioreactor. In this study, we established large-scale fermentation conditions using 10 L and 200 L bioreactors for mass production of *H. chejuensis*-derived prodigiosin, and also found that selective medium optimization can markedly increase the yield of the red pigment. Finally, highly purified prodigiosin from large-scale fermentative *H. chejuensis* cultures was then tested for anticancer activities using human leukemia cancerous cells.

## Materials and Methods

### Cultivation of *H. chejuensis* and Extraction of Prodigiosin at the 250 ml Flask Scale

Marine broth (BD Difco, USA) was used to culture *H. chejuensis* (KCTC 2396) as described previously. If needed, carbon sources (L-arabinose, D-fructose, D-galactose, D-glucose, maltose, D-mannose, and sucrose) were added to marine broth at a final concentration of 0.4%. Cell growth was measured by reading the optical density at 600 nm (OD_600_) using a Libra S70 Spectrophotometer (Biochrom, UK). For prodigiosin extraction, cells and debris were harvested by centrifugation (4,000 rpm, 30 min), and the supernatant was discarded. Next, 100%ethanol (the same volume as that of the culture broth) was added to the collected cells and debris, and was mixed vigorously by vortexing. After ethanol extraction, cell debris was pelleted by centrifugation (4,000 rpm, 30 min) and discarded. The concentration of prodigiosin in the supernatant was measured by high-performance liquid chromatography (HPLC; Agilent 1100 Series HPLC System; Agilent, USA) using a C18 column (WAT05427, 100 Å, 5 μm, 4.6 × 250 mm; Waters Corp., USA). Isocratic elution was performed at 25°C with a flow rate of 0.8 ml/min using a methanol:acetonitrile:distilled water (1:1:2, vol/vol/vol) solution (pH adjusted to 3.6 using acetic acid) as the mobile phase.

### Large-Scale Culture Conditions for *H. chejuensis*

*H. chejuensis* (KCTC 2396) cells were streaked and grown on marine agar (BD Difco) at 30°C. To prepare the initial seed culture, a single colony of *H. chejuensis* was inoculated into 25 ml marine broth (approximate formula per liter, 5 g peptone, 1 g yeast extract, 0.1 g ferric citrate, 19.45 g sodium chloride, 5.9 g magnesium chloride, 3.24 g magnesium sulfate, 1.8 g calcium chloride, 0.55 g potassium chloride, 0.16 g sodium bicarbonate, 0.08 g potassium bromide, 34 mg strontium chloride, 22 mg boric acid, 4 mg sodium silicate, 2.4 mg sodium fluoride, 1.6 mg ammonium nitrate, and 8.0 mg disodium phosphate), and cultured at 30°C for 48 h with shaking at 200 rpm. A portion of the seed culture (2%) was transferred to a 2 L baffled flask and grown on a shaker at 120 rpm and 25°C for 96 h. Large-scale *H. chejuensis* fermentation with added glucose to produce prodigiosin was carried out by batch culture in 10 L and 200 L flasks. After cultivation, bacteria were then harvested by centrifugation at 15,000 rpm for 20 min.

### Purification of Prodigiosin Produced during Fermentation

After the scaled-up production of prodigiosin during fermentation in the 200 L bioreactor, cultured *H. chejuensis* cells were centrifuged at 15,000 rpm for 20 min at 4oC. To maximize the yield, the supernatant and cell pellet were processed separately. Cell pellets were dissolved in 100% ethanol and filtered through a filter paper. The eluent crude extracts were combined with supernatants and evaporated in vacuo in an evaporator. The dried extracts were further purified by preparative HPLC (Hanbon, China), using a machine equipped with the Newstyle NP7000 serial pump (Hanbon) and a dynamic axial compression column (500 × 100 mm (length × diameter)) packed with 100-Ф ODS resin (Daiso, Japan). Glass column chromatography (400 × 115 mm (length × diameter), packed with HP20 resin (Mitsubishi, Japan)) was used to remove impurities. Dried crude prodigiosin was dissolved in methanol with 0.1% formic acid and loaded onto the equipped column. A 40% ethanol solution was loaded onto the column, and free sugar molecules and unwanted hydrophilic compounds from HP20, which were adsorbed by the beads, were washed away with six bed volumes of water, and then samples were eluted with 100% ethanol. Purified prodigiosin was eluted with a retention time of 35–55 min (data not shown). Ethanol-eluted prodigiosin was evaporated *in vacuo*, and its purity was determined by HPLC.

### Analytical Methods

Crude prodigiosin was first extracted with 100% ethanol from a *H. chejuensis* cell pellet obtained by centrifugation (15,000 rpm, 20 min). Its purity was confirmed using an HPLC system (Young Lin Co. Ltd., Korea) and liquid chromatography–mass spectrometry (LC–MS). Mobile phases were 0.1% formic acid in water (solvent A) and methanol (solvent B), and the detection wavelength was 535 nm. HPLC was performed on a YL9120 UV/Vis Detector with a Prodigy ODS-2 column (inner diameter of 150 × 4.6 mm, particle size of 5 μm; Phenomenex, USA) for 20 min with an injection volume of 20 μl crude prodigiosin extract (10 mg/ml). The HPLC chromatogram was compared with that of the pure prodigiosin standard (prodigiosin hydrochloride from *S. marcescens*; Sigma-Aldrich). To further validate the presence and purity of prodigiosin, the extract was subjected to LC–MS performed on a Waters Micromass ZQ Detector using a Prodigy ODS-2 column (inner diameter of 150 × 4.6 mm, particle size of 5 μm; Phenomenex). The column temperature was maintained at 26°C, and mobile phases were 0.1% formic acid in water (solvent A) and methanol (solvent B). The flow rate for loading was 12 L/min. The eluents were then subjected to a capillary voltage of 4 kV, a source temperature of 120°C, and a desolvation temperature of 350°C. Isolated crude prodigiosin was finally purified by subsequent preparative HPLC. Isocratic elution was performed for 80 min (solvent A, double-distilled water; solvent B, 0.1% formic acid in methanol). The elution of high-purity prodigiosin was monitored over a retention time of 30 min using 3.5 g crude extract in 500 ml methanol. After repeated injections, approximately 360 mg purified prodigiosin was isolated from 35 g crude prodigiosin, followed by HPLC and LC–MS analyses.

### THP-1 Cell Culture

The human myelogenous leukemia cell line THP-1 (American Type Culture Collection, USA) was cultured in RPMI 1640 medium (Gibco-BRL, USA) with 10% fetal bovine serum (Hyclone Laboratories, USA), penicillin (100 U/ml), and streptomycin (100 μg/ml) at 37°C with 5% CO_2_ in a humidified incubator. Cells maintained under these conditions were considered undifferentiated, monocytic leukemia cells.

### Measurement of Cytotoxicity and Cell Viability

THP-1 cells were cultured as described in the ‘THP-1 cell culture’ subsection. A Quanti-Max WST-8 Cell Viability Assay Kit (Biomax Ltd., Korea) was used to analyze cell viability according to the manufacturer’s instructions. THP-1 cells (5 × 10^4^ cells per well) were seeded into 96-well plates and incubated overnight. All assays were performed in triplicate. Cells in each well were suspended in 100 μl fresh medium containing various concentrations of prodigiosin, and then were reseeded. After prodigiosin treatment as described above, 10 μl WST-8 was added to each well, samples were incubated for 2 h, and the absorbance at 450 nm was measured.

### Statistical Analysis

Data were analyzed using GraphPad Prism software and reported as mean ± standard error of the mean. Statistical analyses were performed using Student’s *t*-test (for paired or unpaired samples as appropriate). *p*-values < 0.05 were considered significant.

## Results and Discussion

### Production of *H. chejuensis*-Derived Prodigiosin Using Various Carbon Sources

**Fig. 1:**
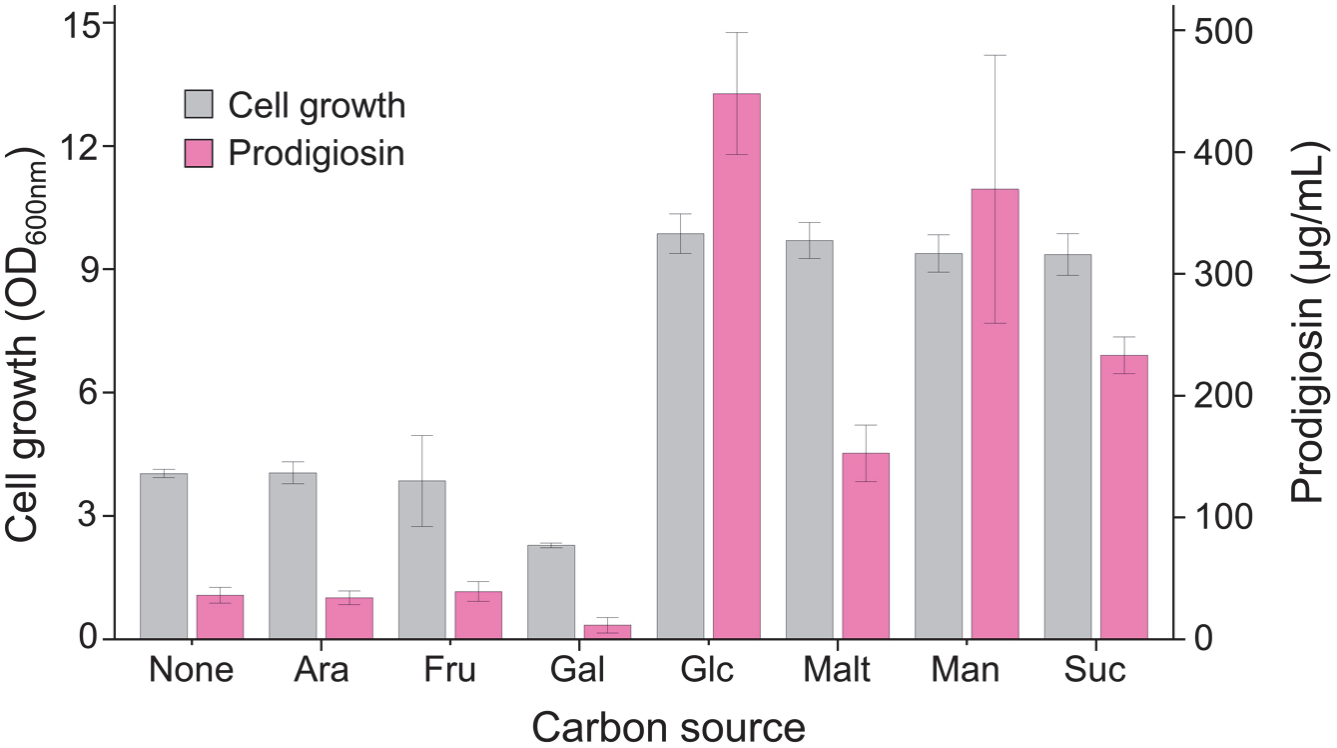
Effect of the addition of various carbon sources (none, marine broth only; Ara, L-arabinose; Fru, D-fructose; Gal, D-galactose; Glc, D-glucose; Malt, maltose; Man, D-mannose; Suc, sucrose) on cell growth and prodigiosin production over 24 h.

Our previous attempt to produce prodigiosin derived from *H. chejuensis* was carried out in the basal marine broth without being investigated to optimize culture conditions [7]. Various carbon sources (L-arabinose, D-fructose, D-galactose, D-glucose, maltose, D-mannose, and sucrose) were added to marine broth to compare cell growth and prodigiosin production for 24 h (Fig. 1) in the presence of different carbon sources. The addition of L-arabinose or D-fructose (final concentration of 0.4%) did not result in any significant change in cell growth or prodigiosin production compared with unsupplemented marine broth, suggesting that *H. chejuensis* cells cannot metabolize L-arabinose or D-fructose. We observed that adding D-galactose affected cell growth and prodigiosin production. When adding D-glucose, maltose, D-mannose, or sucrose, we observed increased cell growth (OD_600_ of 9.6–9.8) and prodigiosin production (152.7–448.1 μg/ml) compared with those from marine broth without any sugars (OD_600_ of 4.0; 36.0 μg/ml). In particular, cell growth and prodigiosin production were increased by 2.4-fold and 12.4-fold, respectively, by adding D-glucose to the culture medium (Fig. 1).

### Scaled-Up Production of *H. chejuensis* Prodigiosin in Bioreactor Systems

**Fig. 2:**
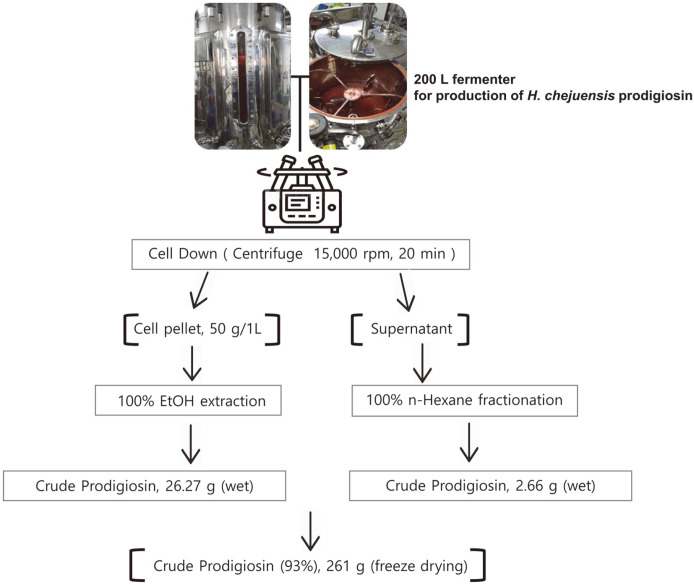
Large-scale prodigiosin production. Prodigiosin was produced by *H. chejuensis* under optimal conditions, and about 50 g/l cell pellet was obtained. 261 g total amount was obtained as freeze-dried crude prodigiosin produced from the optimized cultivated *H. chejuensis*. Finally, prodigiosin purified from fermentation cultures in the scaled-up 200 L bioreactor system was obtained at 99.5% purity.

**Fig. 3:**
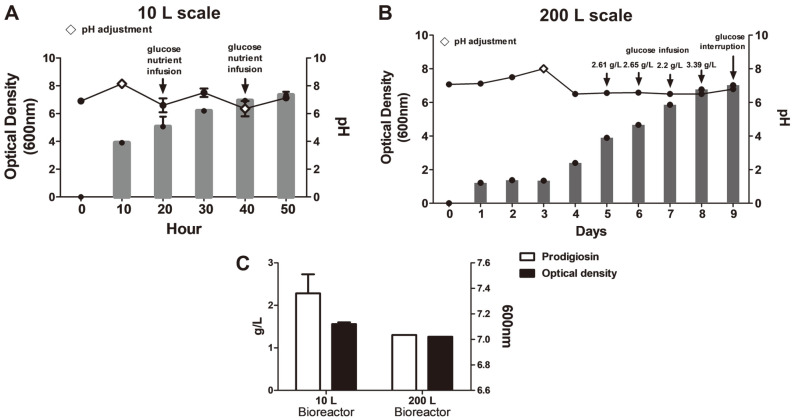
Growth of *H. chejuensis* and changes in culture pH during prodigiosin production using optimally designed medium in the scaled-up condition. The bar graph shows growth from OD_600_ readings, and the line graph shows the pH change in small-scale 10 L (**A**) and large-scale 200 L (**B**) cultures. The lozenge symbol (◇) indicates pH adjustments. Glucose injection or interruption of glucose injection is indicated at each point. (**C**) Prodigiosin production in 10 L and 200 L bioreactor systems for different periods of time at nearly the same optical density.

**Fig. 4:**
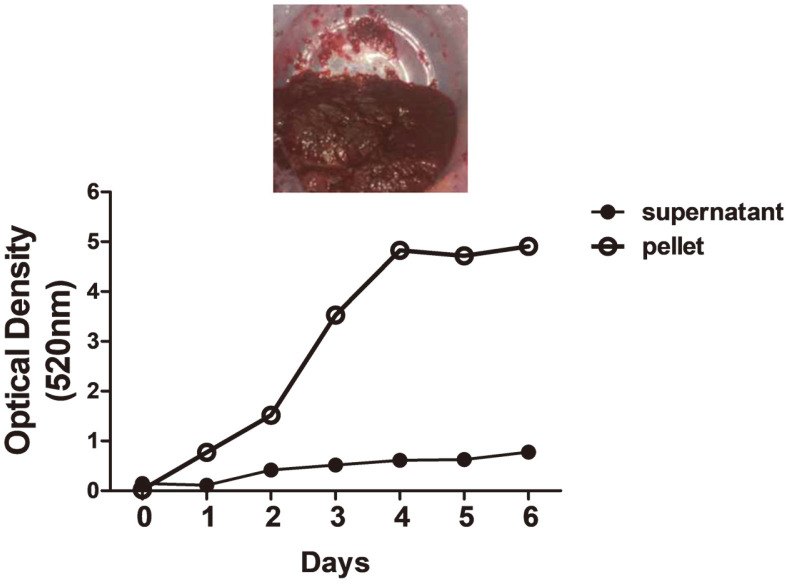
Changes in absorbance of *H. chejuensis* extracts during prodigiosin production in the 200 L pilot fermentor. The line graph shows optical density (520 nm) readings of the ethanol extraction of the red-pigmented pellet and supernatant collected at each time point.

Recently, numerous reports have developed methods to process prodigiosin, mainly derived from *S. marcescens*, with high productivity. However, in most previous studies, prodigiosin was biosynthesized at a small scale, such as culturing in T-flasks using basal marine broth. Based on results from small-scale *H. chejuensis* cultures (sealed T-flask, shaking) stated in the previous section, glucose is a valuable source for increasing prodigiosin production. To scale up prodigiosin production via *H. chejuensis* fermentation, two different sizes (10 L and 200 L stirred-tank reactors with actual working volumes of 5 L and 100 L, respectively) of bioreactor systems were utilized, and, after harvesting the wet cell pellet, the crude extract of the red compound was fractionated in a column loaded with silica gel and finally confirmed to be prodigiosin by HPLC analysis and LC–MS, as briefly shown in a schematic (Fig. 2). The culture temperature was maintained at 37°C, and the pH of growth media was adjusted to 6.8 ± 0.5 by adding 2.5 g/l glucose or 0.1 mM phosphate buffer for metabolite secretion and growth. The pH of cultures of this bacterium increased during growth (Figs. 3A and 3B). Notably, during the exponential growth phase, glucose (concentration of 2.5 g/l) injected once a day was consumed in half a day, and the OD increased. When the fermenting cultures in two bioreactors reached an OD_600_ of 7.5 ± 0.8, cells were collected by centrifugation, and 50 g/l wet cell pellet was harvested and dissolved in 100% ethanol. To verify the production of red-pigmented prodigiosin during *H. chejuensis* fermentation, the OD was additionally measured at the wavelength (520 nm) corresponding to the red dye, followed by sampling at regular intervals during the entire culture period, prior to extraction with 100% ethanol (Fig. 4). Although there have been no reports describing the large scale production of prodigiosin derived from the *H. chejuensis* using 10 L or 200 L bioreactors, studies with various strains have been carried out scale-up of prodigiosin production in bioreactors, as shown in Table 1. Of note, the fermentation time required to harvest the maximum prodigiosin yield in the bioreactors was relatively short or prolonged due to the gene expression variation between different bacterial strains.

### Isolation and Quantification of Prodigiosin from Fermentation Cultures in Large-Scale Bioreactors

**Fig. 5:**
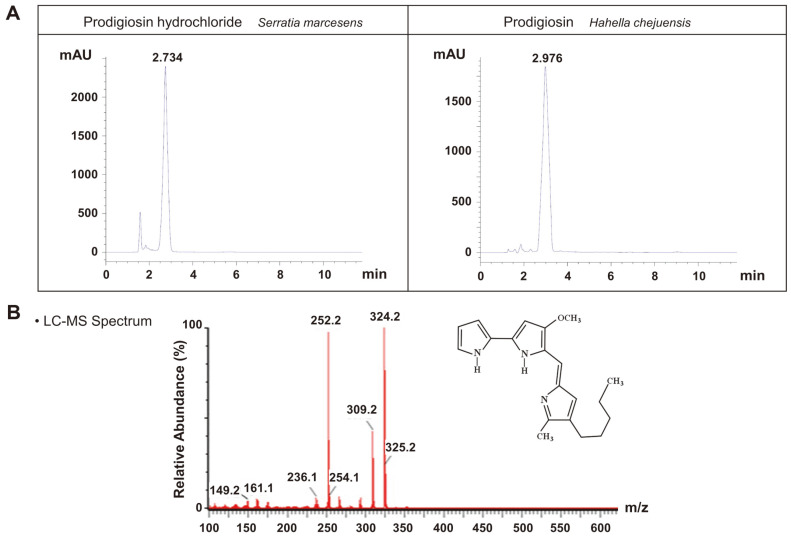
Identification of prodigiosin in the *H. chejuensis* crude extract. (**A**) Prodigiosin extracted from *H. chejuensis* was solubilized in 10 mg/ml DMSO, and the reference value of the sample was compared with the prodigiosin standard from *S. marcescens*. High-purity prodigiosin was detected at a retention time of ~3 min by HPLC. (**B**) The molecular weight of prodigiosin (324.2 g/mol) was determined by LC–MS analysis.

To investigate the physiological role of prodigiosin, we purified a natural crude prodigiosin extract instead of synthetic prodigiosin. In this study, we present a method for prodigiosin production via glucose fermentation. Prodigiosin was first extracted from *H. chejuensis* fermentation cultures. After fermentation, 200 L broth was centrifuged, and the cell pellet was then dissolved in 100% ethanol. HPLC and LC–MS analyses of crude prodigiosin (261 g) showed that prodigiosin was the predominant compound in the crude extract (Fig. S1). To obtain high-purity prodigiosin, additional purification steps were carried out using the crude sample. The maximum yield of high-purity prodigiosin was 36 mg/3.5 g. As a result, approximately 360 mg high-purity prodigiosin (purity, 99.5%) was obtained in total. High-purity prodigiosin was identified by HPLC and LC–MS. As reported for *S. marcescens*, natural red pigment prodigiosin has maximum absorption at 537 nm [20]. Based on analytical results, a single peak from *H. chejuensis*-derived prodigiosin was identified and compared with a prodigiosin standard from *S. marcescens*, and the retention time of purified prodigiosin was identical to that of the standard, as shown in the HPLC profile in Fig. 5A. To confirm the molecular weight of prodigiosin, LC–MS results showed that the highest peak was detected at *m/z* = 324.2 g/mol (M+H)^+^, as shown in Fig. 5B, which corresponds to the expected value for prodigiosin (C_20_H_25_N_3_O). Preparative HPLC analytic profile for high purity of prodigiosin showed that prodigiosin had a retention time of 30 min (Fig. S2). Therefore, the red compound was verified as prodigiosin by HPLC profiling and LC–MS.

Based on our current results, we hope that this study will contribute to optimizing the large-scale production of prodigiosin derived from *H. chejuensis*. The precise spectrum of prodigiosin presented in this report could provide scientific evidence to support potential applications of *H. chejuensis* prodigiosin. Recently, the antimicrobial activity of purified bacterial prodigiosin has been repeatedly reported as valued biological potential [21, 22]. Prodigiosin structure affects antibacterial activity [23, 24], and its strong antifouling properties prevent bacterial biofilm formation [21, 25].

### *H. chejuensis* Prodigiosin Produced by Large-Scale Fermentation Has Cytotoxic Effects on Human Leukemia THP-1 Cells

**Fig. 6:**
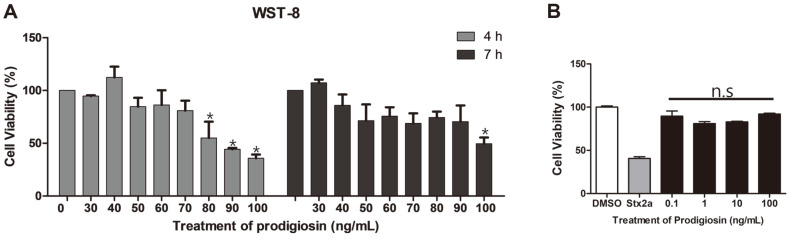
Cytotoxic effects on cancerous human myelogenous leukemia THP-1 cells and non-cancerous renal epithelial Vero cells in the presence of prodigiosin derived from *H. chejuensis* via small- or large-scale fermentation. (**A**) THP-1 human monocytic leukemia cancer cells were maintained in RPMI 1640 medium containing 10% FBS at 37°C with 5% CO_2_ in a humidified chamber. Cytotoxicity in human leukemia cells was measured using the WST-8 assay in the presence of prodigiosin extracted from *H. chejuensis*. (**B**) Cytotoxic effect of prodigiosin in non-cancerous Vero cells. DMSO and Stx2a were used as negative and positive control, respectively. Statistical significance was calculated using Student *t*-test (*p < 0.05), control (no treatment) versus prodigiosin treatments (30 ng/ml to 100 ng/ml); not significant (n.s).

Secondary metabolites derived from natural sources including plants, fungi, or marine bacteria have been used to develop novel potent anticancer compounds. We examined the anticancer effect of prodigiosin in the human myelogenous leukemia cell line THP-1. THP-1 cells were treated for 4 or 7 h with prodigiosin dissolved in dimethyl sulfoxide (DMSO) at various concentrations. To confirm the effect of prodigiosin, we measured the amount of dehydrogenase in live cells using the WST-8 assay. Bacterial Shiga toxin type 2 (Stx2a) was used as a positive control for cytotoxicity in THP-1 cells. Numerous reports showed that Shiga toxins produced by enterohemorrhagic *Escherichia coli* induce programmed cell death in cancerous cells expressing the toxin receptor globotriaosylceramide (Gb3), such as THP-1 cells [26, 27]. Treatment with 30–100 ng/ml prodigiosin for 4-7 h decreased viability in THP-1 cells (Fig. 6A). In contrast to cancerous cells treated with prodigiosin, the non-cancerous kidney epithelial Vero cell line was not susceptible to various doses of prodigiosin (Fig. 6B). These results suggest an anticancer effect because prodigiosin induced cancerous leukemia cell death without killing non-cancer cells. Notably, as described in our previous study [7], in addition to its anticancer effect, crude prodigiosin extracted from *H. chejuensis* reduced levels of ultraviolet-induced reactive oxygen species and the pro-inflammatory response in human keratinocyte cells, revealing potential for developing functional cosmetic ingredients.

In conclusion, we highlight that mass production of *H. chejuensis*-derived prodigiosin could be successfully scaled up from T-flasks to bioreactors of 10 L to 200 L for fermentation with optimized culture conditions to maximize productivity, based on the effects with various carbon sources added in basal marine broth for culturing the bacteria. Moreover, as described in many other studies [1, 5], highly pure (99.5%) *H. chejuensis* prodigiosin via fermentation was beneficial and functionally active, causing cytotoxicity in human leukemia cells whereas not in non-cancerous cells. Furthermore, these results may contribute to the advance for preoccupation in the competition of initiative to secure biotech strategic materials due to the severe effect of the Nagoya Protocol and also contribute to industrialization such as commercialization of active ingredients in beneficial applications.

## Supplemental Materials

Supplementary data for this paper are available on-line only at http://jmb.or.kr.

## Figures and Tables

**Table 1:** Large scale production of prodigiosin in bioreactors from various strains.

Strain	Scale production (L)	Productivity yield (g/l)	Culture time	Ref.
*S. marcescens* TNU01	15	6.20	8 h	[8]
*S. marcescens* TNU01	10	3.45	12 h	[15]
*S. marcescens* TNU02	15	5.10	8 h	[11]
*S. marcescens* 02	5	0.583	20 h	[16]
*S. marcescens*	7	0.595	2.16 days	[17]
*S. marcescens* BS 303 (ATCC 13880)	1.5	0.872	2.7 days	[18]
*Chryseobacterium artocarpi* CECT 849	100	0.522	1 day	[19]
*H. chejuensis* (KCTC 2396)	10	2.28	2.1 days	This study
*H. chejuensis* (KCTC 2396)	200	1.305	10 days	This study
