# Supplementary material for: Enhanced Large-Scale Production of Hahella chejuensis-Derived Prodigiosin and Evaluation of Its Bioactivity
Source: J Microbiol Biotechnol. 2021 Oct 14;31(12):1624–31. doi: 10.4014/jmb.2109.09039 (PMC9705908; doi:10.4014/jmb.2109.09039)
Supplement: Supplementary file 1 [file jmb-31-12-1624-supple.pdf]

J. Microbiol. Biotechnol.
https://doi.org/10.4014/jmb.2109.09039

(A)

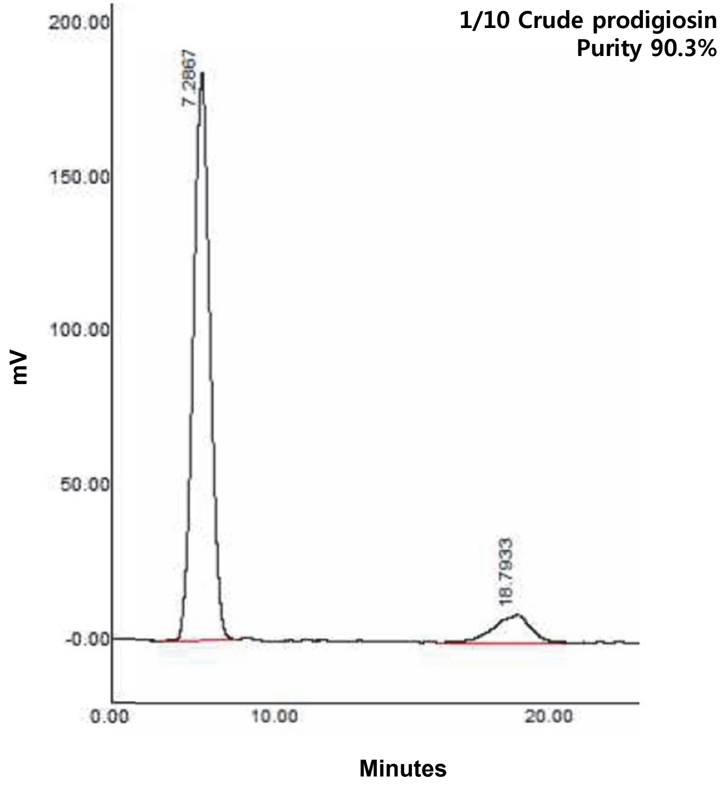

(B)

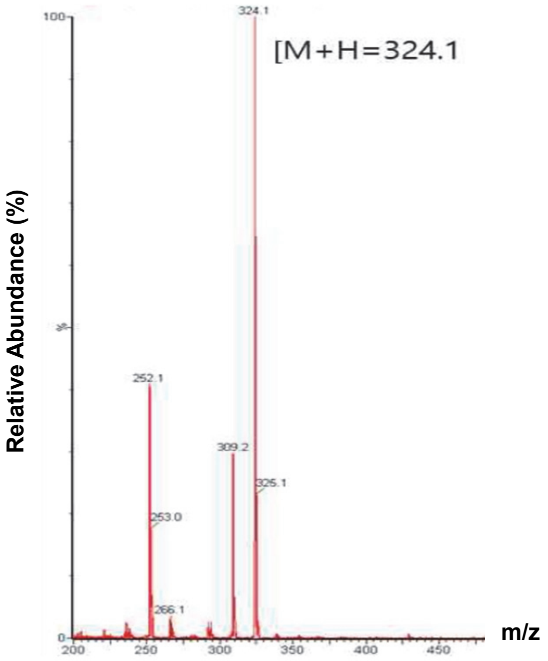

Supplementary Figure 1. (A) Crude prodigiosin is detected at the retention time of ~10 minutes by HPLC. (B) The molecular weight of prodigiosin (324.1 g mol-1) was detected from the liquid chromatography/mass spectrometry (LC-MS) analysis.

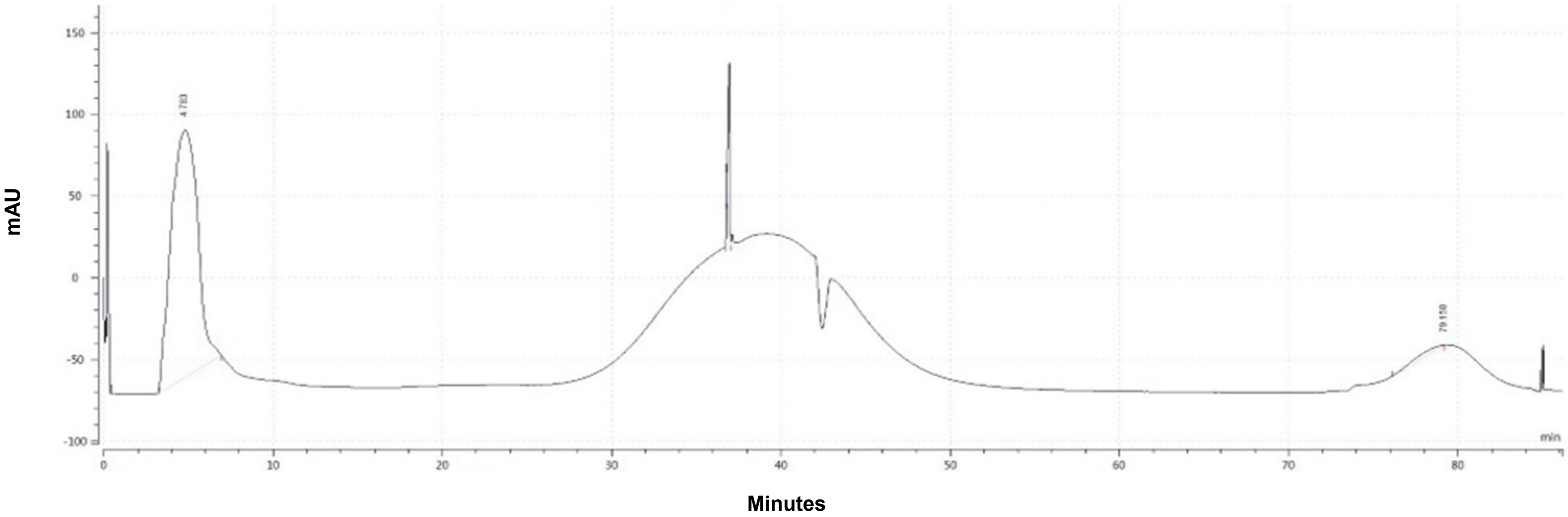

Supplementary Figure 2. Photodiode-Array Detection (PDA) 535nm prep-HPLC results shows that prodigiosin peak was monitored retention time of 30 minutes.
